# A multilevel CPAP support strategy implemented within a large stroke clinical trial (sleep SMART)

**DOI:** 10.3389/frsle.2026.1832143

**Published:** 2026-05-25

**Authors:** Devin L. Brown, Jeffrey S. Durmer, Mark Aloia, Rebecca M. Hankla, Kayla Novitski, Joelle B. Sickler, Shannon Considine, Emerson Delacroix, Jason C. Ong, Alp Sinan Baran, Craig S. Anderson, Dawn M. Bravata, H. Klar Yaggi, Valerie Durkalski-Mauldin, Ronald D. Chervin

**Affiliations:** 1 Department of Neurology, University of Michigan, Ann Arbor, MI, United States; 2 Limbico Health, Denver, CO, United States; 3 Division of Pulmonary, Critical Care and Sleep Medicine, Department of Medicine, National Jewish Health, Denver, CO, United States; 4 Nox Health, Alpharetta, GA, United States; 5 Department of Neurology and Rehabilitation Medicine, University of Cincinnati, Cincinnati, OH, United States; 6 Center for Health Communication Research, Rogel Cancer Center, Michigan Medicine, Ann Arbor, MI, United States; 7 Department of Internal Medicine, Michigan Medicine, Ann Arbor, MI, United States; 8 The George Institute for Global Health, University of New South Wales, Sydney, NSW, Australia; 9 Institute of Science and Technology for Brain-Inspired Intelligence, Fudan University, Shanghai, China; 10 Neurology Department, Royal Prince Alfred Hospital, Sydney, NSW, Australia; 11 Indiana University School of Medicine, Departments of Internal Medicine and Neurology, Indianapolis, IN, United States; 12 Regenstrief Institute, Indianapolis, IN, United States; 13 Department of Internal Medicine, Section of Pulmonary, Critical Care, and Sleep Medicine, Yale School of Medicine, New Haven, CT, United States; 14 Clinical Epidemiology Research Center, Department of Veterans Affairs Connecticut Healthcare System, West Haven, CT, United States; 15 The Data Coordination Unit (DCU), Department of Public Health Sciences, Medical University of South Carolina, Charleston, SC, United States; 16 Department of Neurology and Sleep Disorders Center, University of Michigan, Ann Arbor, MI, United States

**Keywords:** clinical trial, CPAP, CPAP support, sleep apnea, stroke

## Abstract

**Introduction:**

Continuous positive airway pressure (CPAP) clinical trials require integrated CPAP support programs, especially in challenging patient populations. Herein, we describe the CPAP support program devised and implemented within the largest CPAP trial to date in patients with recent acute stroke, the Sleep for Stroke Management and Recovery Trial (Sleep SMART).

**Methods:**

We developed a comprehensive, primarily remote, multi-level and -component automatically-adjusting CPAP (APAP) use support strategy for application across diverse enrollment sites with varied resources. Although many components were pre-planned, some were developed during the conduct of the trial, reflecting innovation and adaption to new technologies. Sites received training and guidance on APAP during the inpatient setting, and a robust telemedicine-based support program was implemented to maximize participant convenience and access. The APAP support program included patient-level behavioral and educational strategies, technical support, objective monitoring and feedback, social support, and system-level facilitation to address the complex determinants of APAP use.

**Results:**

Among the 146 sites across the United States, 138 enrolled at least one participant, and 129 sites randomized at least one participant. Overall, 1,892 participants were equally randomized (1:1) between the two treatment arms from 2019 to 2025, and outcome assessments are ongoing.

**Discussion:**

In this large multicenter clinical trial of APAP in stroke patients, a range of APAP support components were implemented at site- and participant-levels. A comprehensive and standardized APAP support program can be delivered, using a combination of centralized and non-centralized tools, without reliance on local sleep medicine expertise for a clinically complex and difficult-to-treat population.

**Clinical trial registration:**

clinicaltrials.gov, identifier NCT03812653.

## Introduction

Obstructive sleep apnea (OSA) is highly prevalent among patients with stroke (Seiler et al., 2019). The traditional treatment for OSA, continuous positive airway pressure (CPAP), poses challenges for patients with OSA that are amplified among patients with stroke, resulting in high CPAP discontinuation and suboptimal use (Brill et al., 2019). Patients with recent stroke have competing priorities, and often have physical, cognitive, language, or mood-related issues that influence their motivation and ability to use CPAP. Furthermore, stroke patients are often less symptomatic from OSA (Arzt et al., 2010) and thus, may experience less perceived benefit.

Nonetheless, because OSA is associated with worse stroke outcomes and greater recurrence in observational studies (Lisabeth et al., 2019; Brown et al., 2019), and pilot trials suggest possible benefit from CPAP (Sampsonas et al., 2025), definitive trials of CPAP for post-stroke OSA are needed. The success of these trials necessitates implementation of practical CPAP support programs. Herein, we describe the rationale for and implementation of, a comprehensive, remote, multi-level and -component CPAP support strategy within the Sleep for Stroke Management and Recovery Trial (Sleep SMART), the largest post-stroke trial of CPAP to date. Given the lack of a standardized approach to manage CPAP soon after stroke, we present the framework used in this trial to contextualize forthcoming Sleep SMART results, and to inform future trials through documentation of a feasible, scalable approach to predominantly remote CPAP support, implemented across a wide range of hospitals. A full report of the CPAP use data will be presented, once available, with the main trial results.

## Methods

### Context and trial overview

Sleep SMART is a phase 3, investigator-initiated trial that tests whether automatically-adjusting CPAP (APAP) improves stroke recovery or prevention among patients with ischemic stroke or high-risk (ABCD^2^ score ≥ 4) transient ischemic attack (TIA). Eligibility criteria are found in Table 1. Participants with OSA are randomized to 6 months of APAP plus best medical therapy vs. best medical therapy alone. The overall methods have been published previously (Brown et al., 2020), and the trial is registered on clinicaltrials.gov (NCT03812653). The trial is implemented through an NINDS-funded clinical trials network, NIH StrokeNet. The StrokeNet National Coordinating Center at the University of Cincinnati manages contracting, issues site payments, and serves as the central Institutional Review Board (IRB). The StrokeNet National Data Management Center at the Medical University of South Carolina is responsible for data management and data analyses. Overall, 1,892 participants were equally randomized (1:1) between the two treatment arms from 2019 to 2025, and outcome assessments are still ongoing. Of the 146 sites across the United States that were opened at any time, 138 enrolled at least one participant, and 129 sites randomized at least one participant.

**Table 1:** Sleep SMART eligibility criteria.

Inclusion criteria
Age ≥18 years
Ischemic stroke within the prior 7 days with an NIHSS ≥ 1, or ischemic stroke or high risk TIA (ABCD^2^ ≥ 4) within the prior 14 days (depending on time period). The ischemic stroke definition was based on clinical criteria and did not require an area of acute infarction on neuroimaging.
Exclusion criteria
1. Pre-event inability to perform all of own basic ADLs
2. Unable to obtain informed consent from subject or legally authorized representative
3. Incarcerated
4. Known pregnancy
5. Current mechanical ventilation (can enroll later if this resolves) or tracheostomy
6. Current use of positive airway pressure, or use within 1 month prior to stroke
7. Anatomical or dermatologic anomaly that makes use of CPAP interface unfeasible
8. Severe bullous lung disease
9. History of prior spontaneous pneumothorax or current pneumothorax
10. Hypotension requiring current treatment with pressors (can enroll later if this resolves)
11. Other specific medical circumstances that conceivably, in the opinion of the site PI, could render the patient at risk of harm from use of CPAP
12. Massive epistaxis or previous history of massive epistaxis
13. Cranial surgery or head trauma within the past 6 months, with known or possible CSF leak or pneumocephalus
14. Recent hemicraniectomy or suboccipital craniectomy (i.e., those whose bone has not yet been replaced), or any other recent bone removal procedure for relief of intracranial pressure
15. Current receipt of oxygen supplementation >4 liters per minute
16. Current contact, droplet, or respiratory/airborne precautions

Patients were recruited during hospitalization for an acute stroke/TIA or just-subsequent rehabilitation, during which initial OSA testing was conducted, followed by an APAP run-in night if OSA but not significant central sleep apnea was present. As local sleep medicine expertise is not routinely available for inpatients, and approaches to such care could vary between sites, Sleep SMART was designed to operate without it. Stroke teams were asked to implement the protocol with the assistance of inpatient respiratory therapists, sleep technologists, or trained coordinators. After discharge, APAP nationwide care management was provided remotely via telemedicine by Nox Health (formerly FusionHealth). To ensure consideration of all viable but generalizable options to promote APAP use, APAP support was periodically reviewed with the Sleep SMART Steering Committee, including a CPAP Support Subcommittee. A study consultant (MA) who is a psychologist with international recognition for research and expertise in CPAP use, also provided input, guidance, and training. Although nightly use for the entire night was the goal in Sleep SMART, we emphasized with participants that CPAP use can be challenging, and that small improvements in CPAP use should be celebrated.

The Nox T3^TM^ sleep apnea test was used in accordance with the intended use to identify OSA based on standard diagnostic and scoring methodologies (Brown et al., 2020). The test could be repeated if at least 3 h of usable data were not collected. Tests included a nasal pressure cannula, two respiratory inductance plethysmography (RIP) belts, ECG leads, and pulse oximetry unit. Devices were applied by study teams and data were uploaded the following day. Studies were scored centrally by Nox Health sleep technologists who edited recordings for start and stop time, eliminated artifacts, and applied automated scoring using Noxturnal. When nasal pressure was missing or not reliable, RIPflow derived from the two calibrated RIP belts (Montazeri et al., 2021) was used to estimate apneas and hypopneas, as recommended (Berry et al., 2017). OSA was defined as a respiratory event index (REI) ≥ 10 events/h where the central apnea index (CAI) comprised less than half of the REI. Site teams remained masked to the sleep apnea test report.

Participants with qualifying OSA underwent a single APAP run-in night using the ResMed automatically-adjusting AirSense^TM^ 10 or 11. To be eligible for randomization, usage during the run-in night had to be ≥4 h with the machine-estimated CAI (number of central apneas per hour of machine use) <10. The participant also had to express continued comfort, after the run-in night, with 1:1 randomization, meaning a 50% chance of APAP use for 6 months and 50% chance of no CPAP (care as usual) for 6 months. The run-in night could be repeated up to two times (three total run-in nights) if use was <4 h as a result of clinical activities, or a reason not likely to be repeated. As added to the protocol during the trial, the run-in night could be repeated once for any reason if the participant was amenable and the CAI on the previous attempt was <10. Inclusion of only participants able to use APAP for at least 4 h during the run-in night was anticipated to focus the trial on participants most likely to achieve considerable APAP use at home.

### APAP support program components

Many elements of the program were pre-planned in collaboration with Nox Health, based on their experience in previous research and current sleep telehealth programs in which high CPAP use is a key quality measure of value-based care (Riney et al., 2025).

Other components of the program were developed based on experience gained during the nearly 7-year execution of Sleep SMART. Changes in strategies were proposed and considered based on continuing input from multiple sources, including participants, clinical research coordinators, a clinical research coordinator advisory group, Nox Health sleep coaches and its collaborating investigators, the CPAP consultant mentioned above, and experts in motivational interviewing. Strategies in Sleep SMART—pre-planned and innovated during its execution—were shared via email with sites, IRB amendments when appropriate, and with site clinical coordinators and other investigators who attended monthly webinars and an in-person investigator meeting, where emphasis was placed on the importance of early (inpatient) success in APAP use to long-term ability to achieve high CPAP use (Van Ryswyk et al., 2019; Jeppesen et al., 2025; Villa Alvarez et al., 2022; Jordan et al., 2014).

### Mask selection and acclimation for the run-in night

Preparation for the run-in night could be performed by a respiratory therapist, sleep technologist, or trained study team member. Study team members were provided with how-to videos, written guides, and hands-on workshops. Clinical enrollment sites were originally stocked with three mask types: ResMed N20, P10, and F20 to provide nasal triangle, nasal pillows, and full face mask options for each participant. The Respironics DreamWear was later added as an option for some sites. The FDA issued its first recall in 2022 regarding magnet-containing masks (FDA, 2022). We then phased out the N20, F20, and DreamWear and instead started new participants on the ResMed N30i nasal cushion, P10 nasal pillows, or the Fisher & Paykel Evora full face mask.

In the beginning of the trial, participants tried all three mask types at a fitting session before the run-in night, and selected their preference. Later, participants were shown all three mask types in the packaging and were asked to select a preference to try first, and use if it seemed effective and comfortable. If no preference was expressed, the nasal or nasal cushion mask was selected to be tried first. We discouraged sites from using full face masks as the initial mask, as they are, in comparison to nasal masks, associated on average with lower use, higher pressure requirements, and higher residual AHI (Genta et al., 2020). Chin straps were made available at sites for use if participants continued to mouth-breathe on APAP. Due to conceivable safety concerns, full face masks were not allowed for participants with decreased mental status, on tube feeds, or unable to remove the mask without assistance.

Sites were instructed to provide a 15–20-min daytime trial of APAP to participants prior to the run-in night. This was an opportunity to troubleshoot and acclimatize. Later in the trial, we provided instructions about how to teach participants abdominal breathing as a relaxation technique, and asked sites to have participants use abdominal breathing as they were gradually introduced to APAP (Means and Edinger, 2007). First participants tried the mask alone, and then the mask was connected to the running APAP machine. Toward the end of the daytime trial, fit was checked using the mask fit setting on the APAP machine. We also asked the sites to assist the participant in practicing placement and removal of the mask, repeatedly, until proficiency was reached. This was intended to improve participants' perceived competency with APAP.

Admission to a sleep lab or research unit, and at-home run-in nights were allowable when needed, but infrequently used. Supplies and procedures were the same regardless of the setting.

### Device settings

The AirSense S10 and S11 with heated humidification were used for run-in nights, in-hospital treatment, and after hospital discharge. Initial settings were: AutoSet mode, pressure 5–20 (or 4–20) cm of water, expiratory pressure relief turned “on” full time at level 3, ramp time on auto, SmartStart/SmartStop “on,” humidity level 5, and ClimateLine tubing temperature on auto. We requested that sites not alter the settings on the run-in night devices to standardize the APAP presentation and maximize comfort.

### In-hospital use after the run-in night

Participants randomized to the APAP arm were provided their own APAP device to use during the remainder of the hospitalization and 6-month treatment period. We recommended that the sites arrange overnight support for APAP use by their respiratory therapists or sleep technologists per their usual protocols for patients starting APAP. We suggested that coordinators check in with participants daily to encourage APAP use, and troubleshoot if a respiratory therapist was not doing so. We also suggested that the coordinator ask the clinical inpatient team to provide encouragement.

### CPAP education and preparation for outpatient use

Early in the trial, scripts were drafted for coordinators, to ensure that participants received clear information about OSA, APAP, and the connection between OSA and stroke outcomes. A respiratory therapist, sleep technologist, or trained study team member gave education and training on APAP use to the participant, and partner or family member if present. Publicly-available videos from manufacturers on APAP and mask use were also made available to supplement the teaching. Sites were instructed to show a study-specific video about the outpatient sleep-coaching program provided to participants so they would know what to expect. Coordinators were also asked to assist participants in setting up the CPAP-use tracking app, myAir^TM^ (Malhotra et al., 2018). The no-fee myAir^TM^ app was available to each participant and offered an opportunity for daily feedback. The myAir^TM^ dashboard contained information about usage hours, mask seal, estimated apneic events per hour, and number of mask on/off instances including an overall nightly score to reflect the level of success achieved. Instructional videos were available through the app, which also sent motivational time-based and event-based messages by email to participants. Text messages were also available if selected. Before hospital discharge, a behavioral contract (Supplementary material) was offered to participants to sign and to have a witness sign. This was intended to deepen the participants' commitment to trying APAP to the best of their ability.

Coordinators were instructed to help participants who agreed to place the sleep coach's phone number in their cell phones, so incoming calls would be recognizable. We also asked coordinators to facilitate a call between the sleep coach and participant prior to discharge, to make introductions and initiate a relationship. Each participant was to be notified that if they completed another call with the sleep coach within a week after discharge, they would receive a $10 gift card.

For participants discharged to a care setting other than home, sites were to provide an APAP order to help facilitate APAP use at the rehabilitation facility or nursing home. Templated orders (see Supplementary material) were provided to sites in case local order templates were not available. Templated letters (see Supplementary material) for sites to introduce rehabilitation facilities to Sleep SMART were also provided.

### At the time of hospital discharge

Participants were sent home with their APAP and supplies, a magnet with their sleep coach's telephone number, mask instructions, three one-page instruction sheets, and the link to the study's patient-facing website. The website contained the electronic version of the three instructional sheets, a Sleep SMART care management welcome video; the myAir^TM^ link; a CPAP user guide, and video links for CPAP set up and mask use; and a recap document with reminders about their sleep apnea status, myAir^TM^ app URL, sleep coach phone number, website, and information about a Sleep SMART chatbot (when available, see below).

### Post-hospitalization APAP support and sleep coaching

After index hospital discharge, Nox Health provided APAP support to participants via telemedicine. Sleep coaches proactively monitored participants' APAP use data wirelessly, using AirView^TM^, and used a variety of communication methods (regular phone calls, text messages, secure video calls) to engage participants, review progress, and troubleshoot technical issues. Secure video calls were offered to participants to establish rapport and assist with troubleshooting mask and APAP problems (e.g., mask leaks, high residual AHI). Sleep coaches reviewed the Nox T3 summary results with participants and, when possible, showed oximetry tracings via text or video. When necessary, technical issues were escalated to a respiratory therapist and/or reviewed with a sleep medicine physician and medical issues were escalated to a sleep medicine physician for review (Riney et al., 2025). Sleep coaches also encouraged myAir^TM^ use by the participant, and offered to assist with technical issues.

To enhance intrinsic motivation, sleep coaches employed motivational interviewing techniques, using an autonomy-supportive approach to explore ambivalence and elicit participant-identified goals related to APAP use. The goal was for all contact between the sleep coach and participants to be grounded in motivational interviewing. Coaches received structured training in motivational interviewing, and follow-up training sessions. Roadmaps and standard operating procedures were used that incorporated an explore, guide, and choose framework. Some participant calls were recorded, with the participant's permission, and select calls were reviewed by experienced motivational interviewing instructors for quality assurance and retraining purposes. Calls were scored using the OnePass system to assist with feedback, and to support motivational interviewing fidelity (McMaster and Resnicow, 2015). Sleep coaches also offered to have participants' “CPAP partners” join the calls, or to call the partners separately. A CPAP partner could be a bedpartner, other family member, or other person who knew the participant was trying to use CPAP.

Nox Health staff were able to make a wide array of alternative masks available for participants who had difficulty with their current mask. Early in the trial, it was common for participants to receive 1 to 5 additional masks to try. The frequency of multiple mask changes diminished during the trial, likely related to intentional reduction of masks offered due to cost concerns and the lack of a perceived additional benefit. Nox Health also sent updated supplies such as new tubing and mask cushions as needed. Under the guidance of a Nox Health sleep medicine physician, sleep coaches could recommend over-the-counter treatments (e.g., saline nasal spray), initiate a desensitization process, make device setting adjustments, or switch to bilevel PAP.

Specific criteria prompted Nox Health to request that the trial site offer the participant a referral to a local sleep medicine physician for in-person clinical care. Criteria included ongoing low APAP use, excessive residual AHI, excessive CAI, emergence of other sleep disorders, or clues that another device type (aside from CPAP or bilevel PAP) might be indicated.

Soon after randomization, participants were mailed a thank-you note (see Supplementary material), for trial participation and for use or attempts to use APAP. The letter emphasized the importance of their study participation. A templated letter (see Supplementary material) was provided to sites to send participants who were having significant difficulty using APAP. The information in the letter also served as talking points for a site investigator to use in discussion with the participant.

### Other strategies attempted, but discontinued

Sleep SMART offered several types of support to sites and participants on a trial basis. Clinical research coordinators at selected highly-active sites were asked to contact a central Sleep SMART board-certified sleep medicine physician whenever a participant was randomly assigned to the APAP arm. The physician offered support to coordinators to maximize the chances of success as the participant began inpatient use of APAP. Central Sleep SMART sleep medicine physicians also called participants who were difficult to reach by the sleep coach, or had low APAP use. At other times, coordinators facilitated calls between hospitalized participants and Sleep SMART sleep medicine physicians who then gave an introduction to OSA and APAP, reviewed the purpose of the trial, emphasized resources to help individuals succeed with regular APAP use, and expressed appreciation for their participation. Each of these strategies was tried only temporarily because anecdotal feedback or preliminary data did not suggest they were helpful.

As some participants were not eager to have telephone conversations, for a time, a commercially-available artificial intelligence-driven chatbot, originally designed in part to support mental health, was custom-programmed to accommodate Sleep SMART-specific questions and responses, mainly about APAP. The chatbot was made available to interested participants via text-messaging. The chatbot used a non-directive, autonomy-supportive communication style that acknowledged and reassured users that difficulties with APAP are common. Encouraging messages were sent about APAP use, and trial participation more generally. The chatbot was able to troubleshoot basic APAP issues, and could refer the participants to their sleep coaches. The chatbot was retired due to limited engagement by participants, and only partial uptake by study coordinators.

## Discussion

This report highlights the range of APAP support components implemented within a large, multicenter, randomized controlled trial of CPAP for stroke patients with OSA. It illustrates how comprehensive and uniform APAP support can be devised, using a combination of centralized and non-centralized tools, without reliance on local sleep medicine expertise for a clinically complex, difficult-to-treat population. The APAP support program included patient-level behavioral and educational interventions, technical support, objective monitoring and feedback, social support, and system-level facilitation, to address the complex determinants of CPAP use. This approach reflects the complexity of APAP use behavior that is influenced by behavioral, educational, technical, and system factors. Consistent with a pragmatic approach, we adapted the APAP support program throughout the trial to address newly identified challenges and incorporate evolving technology, while preserving the core components and behavioral targets of the intervention.

Although the APAP support program was developed pragmatically to maximize use within a clinical trial, its components align closely with established behavioral theory. When mapped to the COM-B framework of behavior change, the program components addressed all major domains including capability, opportunity, and motivation (Table 2) (Michie et al., 2011). Capability was supported through education, skills training, technical support, device optimization, and anxiety-reduction strategies. Opportunity was enhanced through reduction of logistical and systems-level barriers, support through telemedicine and digital tools, and engagement of partners and clinical teams. Motivation was addressed through motivational interviewing, reinforcement, and feedback. Across components, the intervention emphasized normalization of early difficulty and progressive mastery of skills, supporting self-efficacy and habit formation rather than immediate perfection.

**Table 2:** Mapping of CPAP support interventions to COM-B domains.

COM-B domain	Targeted behavioral determinants	CPAP Support Interventions
Capability (psychological and physical)	Knowledge and skills; comfort and anxiety management; self-efficacy	• Patient education on OSA, CPAP, and stroke–OSA relationship
		• Instructional “how-to” videos and written materials
		• Gentle introduction to CPAP and mask use using relaxation techniques
		• Daytime trial to allow acclimation and adjustments
		• Mask/interface selection with patient input; multiple mask options
		• Use of state-of-the-art CPAP device with comfort features
		• Technical and troubleshooting support
		• Partner education to reinforce understanding and use
Opportunity (physical and social)	Access, convenience, environmental support, reduced friction	• Telemedicine support for CPAP management
		• CPAP use tracking app (myAir^TM^)
		• Chatbot for troubleshooting and text-based support
		• Patient-facing website with centralized resources
		• Templates for CPAP orders for discharge to rehab or nursing facilities
		• Templated clinician encouragement letters
		• Engagement of partners and clinical teams
		• Involvement of sleep medicine physicians when needed
		• Expert consultation to support intervention design and site implementation
Motivation (reflective and automatic)	Values and goals (reflective motivation); reinforcement, accountability, and habit formation (automatic motivation)	• Motivational interviewing delivered by trained sleep coaches to participants and partners
		• Expectation-setting videos introducing sleep coaches
		• Motivational emails (via myAir^TM^) and chatbot messages
		• Behavioral contract for CPAP use
		• Feedback on CPAP use via tracking app (myAir^TM^)
		• Monetary incentive for initial post-discharge call with sleep coaching
		• Thank-you notes emphasizing contribution and meaning (“hero” framing)
		• Normalization of early difficulty and emphasis on progressive mastery

Other CPAP support programs for stroke patients with OSA have mostly focused on single rather than multiple components. Two single-center trials of telemedicine management showed some benefit for remote monitoring with telephone support calls. The calls were intended to troubleshoot issues and provide encouragement, but behavioral science techniques were not described (Nilius et al., 2019; Kotzian et al., 2019). Another multicenter trial among patients with recent stroke failed to show benefit of a behavioral intervention that focused on barriers and facilitators to CPAP use (Bravata et al., 2018).

In one study after stroke, an educational intervention did not result in greater APAP use (Dharmakulaseelan et al., 2019), while a single-arm, single site study achieved reasonable CPAP use with a 3-component intervention that included motivational interviewing (Khot et al., 2019). A variety of barriers to APAP use among patients have been identified, including low self-efficacy, common APAP-related complaints, limited expected effect of treatment, discomfort with new technology, lack of social support, and need for APAP-related skills (Yu et al., 2025; Khot et al., 2022). We attempted to address these and other barriers through a multifaceted CPAP support program that included motivational interviewing. In the general OSA population, behavioral interventions such as motivational interviewing result in the largest improvement in APAP use; supportive interventions result in a modest improvement; and educational interventions have unclear effects (Askland et al., 2020). Cognitive behavioral therapy for insomnia prior to initiation of CPAP (Sweetman et al., 2019) and peer support through interactive voice response (Parthasarathy et al., 2025) are emerging interventions to improve CPAP use.

Our study had many strengths. Sleep SMART was designed to support APAP initiation and use in a manner relevant to real-world clinical practice, where resources such as local expertise, staffing, insurance coverage, and access to specialized sleep centers vary. The remote-focused model enabled uniform implementation across more than 100 sites, improved scalability, and enhanced access for participants with mobility, transportation, or geographic barriers. This approach is supported by a growing literature showing that telemedicine-based CPAP management improves use (Niu et al., 2023; Verbraecken et al., 2025), including among stroke patients (Nilius et al., 2019; Kotzian et al., 2019). We incorporated evidence-based interventions where possible, innovative support, and at times support not typically available in routine care, including near-real-time monitoring of nightly use, proactive outreach by sleep coaches, structured motivational interviewing, and rapid trials of multiple mask interfaces. These features allowed individualized, responsive support while maintaining consistency across a large multicenter trial.

This study also had some limitations. As the APAP support program was not the intervention under evaluation in the trial, we did not systematically assess its fidelity, including implementation of site-level components or participant engagement. The APAP support intervention evolved over the course of the trial in response to emerging challenges and advances in technology. While this adaptive approach enhanced generalizability to clinical settings where APAP support strategies must evolve in response to patient needs, it introduced some heterogeneity in participant exposure. The study was not designed to evaluate the effectiveness of individual program components or the combined components, precluding attribution of CPAP use metrics to any intervention component. In addition, some newly introduced components were discontinued based on informal feedback and study team experience rather than prespecified criteria, introducing subjectivity into intervention refinement. As APAP has not yet been proven to improve stroke-related outcomes—and this was the basis for equipoise in this randomized trial—we were unable to encourage APAP use based on the anticipated clinical benefit of importance to the participants. In the future, more definitive proof of benefit may positively influence CPAP uptake among stroke patients. Lastly, the largely remote approach to CPAP support excluded some potential in-person CPAP support interventions. Our pragmatic approach involved a tradeoff between scalability and standardization and provision of comprehensive in-person care. In Sleep SMART, participants rarely if ever had a clinic visit with a sleep medicine physician, in-lab PAP titration study, in-person appointments at a durable medical equipment company, PAP-Nap studies, or opportunity to see a sleep psychologist to address barriers to use (Bertrand et al., 2022; Krakow et al., 2008; Ulibarri et al., 2020; Goh et al., 2025; Parmaksiz, 2021; May et al., 2023).

In short, we describe the implementation of a comprehensive, mainly remote, multilevel APAP support program within a large, multicenter trial of APAP among stroke and high-risk TIA patients with OSA. Developed pragmatically to address use challenges in this clinically complex population, and equitably to allow broad participation at a wide variety of medical centers, the program integrated centralized and site-based resources, behavioral support, and technology-enabled tools without reliance on local sleep medicine expertise. The practical framework that emerged may offer considerations for future clinical trials and care models that seek to optimize APAP use among the many patients with recent stroke who also have OSA.
