# Supplementary material for: A multilevel CPAP support strategy implemented within a large stroke clinical trial (sleep SMART)
Source: Front Sleep. 2026 May 25;5:1832143. doi: 10.3389/frsle.2026.1832143 (PMC13243104; doi:10.3389/frsle.2026.1832143)
Supplement: Supplementary file 1 [file Supplementary_file_1.docx]

Date:

**Health contract**

I have enrolled in a research study called Sleep SMART. The purpose of this study is to test whether CPAP improves stroke recovery and prevention. I have been assigned to use CPAP for the next 6 months.

**My ultimate goal is to use CPAP all night, every night for the next 6 months.**

**To reach my ultimate goal, I commit to using CPAP the best I can on most nights of the week, first focusing on the next 3 months.**

**As part of this process, I agree to be in regular contact with my sleep coach.**

If I find regular CPAP use challenging, I agree to reach out to my sleep coach. I know that they can be reached at xxx-xxx-xxxx, and that resources are available to me on this website:

[https://www.nihstrokenet.org/trials/sleep-smart-trial/patients.](https://www.nihstrokenet.org/trials/sleep-smart-trial/patients) I know that I can track my own CPAP use through the myAir app or website.

Participant signature

Witness

**Order for Automatically Adjusting Continuous Positive Airway Pressure (aCPAP)**

**Patient Name:**_____________________ **Date of Birth**: (mm/dd/year):__________________

**Dx:** obstructive sleep apnea G47.33

**Duration:** 6 months

**Device:** ☐ ResMed AirSense 10 AutoSet	☐ ResMed AirSense 11 AutoSet

**Minimum Auto CPAP pressure cm of water:** 5

**Maximum Auto CPAP pressure cm of water:** 20

**Pressure relief:** Yes

**Supplemental oxygen flow:**

☐ No Oxygen ☐0.5 LPM ☐1LPM ☐2LPM ☐3LPM ☐4LPM

**Heated humidification with humidifier chamber:** Yes

**Mask:**

☐Full face mask	☐Nasal/nasal cushion mask	☐Nasal pillows

**Tubing:** ☒Heated (ResMed ClimateLine/ClimateLineAir)

**Additional supplies:**

☐Chinstrap	☒Headgear

**Special instructions: _________________________________________________________**

**Letter to introduce rehabilitation facilities to Sleep SMART**

<Date>

<Rehabilitation Center Medical Director>

Dear Dr. <name>,

I write to introduce an exciting NIH-funded research study in which we at <hospital name> participate. The study, Sleep for Stroke Management and Recovery Trial (Sleep SMART) is being implemented at over 100 hospitals across the United States and plans to enroll thousands of patients. This landmark study tests whether continuous positive airway pressure (CPAP) improves stroke recovery and secondary prevention. As you know, post-stroke sleep apnea is highly prevalent (~75%). It is associated with poor functional and cognitive outcomes, and increased risk of recurrent stroke and mortality after ischemic stroke. In Sleep SMART, after the diagnosis of sleep apnea is made and tolerability of CPAP is confirmed, subjects are randomized to 6 months of CPAP plus usual care, or usual care alone.

Some patients whom we refer to your center for inpatient rehabilitation will be enrolled in Sleep SMART. Intervention arm subjects will already have with them their own study-provided CPAP devices and supplies. The CPAP device automatically adjusts, on a breath-by-breath basis, to provide the appropriate pressure. We will provide an accompanying CPAP order for these subjects, and we ask that they use their own CPAP device while at your center and after they leave.

If you have any questions or concerns, please do not hesitate to phone me at <phone number>.

Thank you for your consideration.

Sincerely,

<name>

**Thank you card text**

You chose to make a difference!

Thank you for your participation in Sleep SMART!

You joined thousands of other stroke or TIA volunteers around the country to participate in a research study. Your experience with CPAP, and information about your recovery will help physicians learn how to offer the best care for stroke patients.

The results of Sleep SMART could improve healthcare in the future for you and for many others as well. Given that both stroke and sleep apnea often run in families, Sleep SMART results could end up helping people close to you in the years to come.

Thanks again for using CPAP and helping with Sleep SMART. You are our hero!

If you haven’t recently, please call your Sleep SMART sleep coach at **xxx-xxx-xxxx** to check in.

**Letter for participants having diffulty with CPAP**

[date]

Dear [participant name],

Thank you for choosing to participate in Sleep SMART.

We understand that you have had issues with your CPAP. Some people don’t take to CPAP right off the bat. It sometimes takes time to get used to CPAP, and adjustments are sometimes needed.

**We know that you have obstructive sleep apnea**. Obstructive sleep apnea happens when your throat closes off causing you to stop breathing. This happens many times per hour during sleep. People with sleep apnea often don’t realize that they stop breathing. Sleep apnea can cause your blood oxygen level to drop many times per hour, and can disrupt your sleep. **Sleep apnea puts people at risk for another stroke, and is linked to worse recovery after stroke.**

Continuous positive airway pressure (CPAP) restores your breathing during sleep. CPAP is considered safe and effective. However, **CPAP can only work to treat sleep apnea when you use it**. The trick is getting used to using it, and then remembering to use it whenever you sleep.

**Fortunately, in Sleep SMART, you are not alone.** Your Sleep SMART sleep coach stands ready to help. The sleep coaching team has supported thousands of people from all over the US using CPAP to treat sleep apnea. **They will tailor your treatment for comfort and effectiveness**. Your sleep coach can provide you with different masks along with other equipment to make your CPAP use easier and more comfortable.

We know that little improvements are a big deal when you’re just starting out, and we’re not looking for perfection. **Right now,** you might find that the one thing you really can control IS your sleep. And by treating your sleep apnea, you can take back the healthy and restful sleep you may have been missing for years.

So, please give a call to your Sleep SMART sleep coaching team at **xxx-xxx-xxxx. They are ready to help.** Please also feel free to visit our website and watch the Sleep SMART Care Management video: [https://www.nihstrokenet.org/sleep-smart-](https://www.nihstrokenet.org/sleep-smart-trial/patients) [trial/patients](https://www.nihstrokenet.org/sleep-smart-trial/patients).

Sincerely,

[signature]
